# Orthologs of *Plasmodium* ICM1 are dispensable for Ca^2+^ mobilization in *Toxoplasma gondii*

**DOI:** 10.1128/spectrum.01229-24

**Published:** 2024-08-20

**Authors:** Gabriel Cabral, Bingjian Ren, Hugo Bisio, Dawson Otey, Dominique Soldati-Favre, Kevin M. Brown

**Affiliations:** 1 Department of Microbiology and Immunology, University of Oklahoma Health Sciences Center, Oklahoma City, Oklahoma, USA; 2 Department of Microbiology and Molecular Medicine, University of Geneva, Geneva, Switzerland; 3 Aix-Marseille Université, Centre National de la Recherche Scientifique, Information Génomique & Structurale, Marseille, France; University of Georgia, Athens, Georgia, USA

**Keywords:** *Toxoplasma gondii*, *Toxoplasma*, *Plasmodium*, calcium, calcium flux, calcium signaling, cyclic GMP, cGMP, cAMP, motility, Apicomplexa, apicomplexan

## Abstract

Apicomplexan parasites mobilize ionic calcium (Ca^2+^) from intracellular stores to promote microneme secretion and facilitate motile processes including gliding motility, invasion, and egress. Recently, a multipass transmembrane protein, ICM1, was found to be important for calcium mobilization in *Plasmodium falciparum* and *P. berghei*. Comparative genomics and phylogenetics have revealed putative ICM orthologs in *Toxoplasma gondii* and other apicomplexans. *T. gondii* possesses two ICM-like proteins, which we have named TgICM1-L (TGGT1_305470) and TgICM2-L (TGGT1_309910). TgICM1-L and TgICM2-L localized to undefined puncta within the parasite cytosol. TgICM1-L and TgICM2-L are individually dispensable in tachyzoites, suggesting a potential compensatory relationship between the two proteins may exist. Surprisingly, mutants lacking both TgICM1-L and TgICM2-L are fully viable, exhibiting no obvious defects in growth, microneme secretion, invasion, or egress. Furthermore, loss of TgICM1-L, TgICM2-L, or both does not impair the parasite’s ability to mobilize Ca^2+^. These findings suggest that additional proteins may participate in Ca^2+^ mobilization or import in Apicomplexa, reducing the dependence on ICM-like proteins in *T. gondii*. Collectively, these results highlight similar yet distinct mechanisms of Ca^2+^ mobilization between *T. gondii* and *Plasmodium*.

**IMPORTANCE:**

Ca^2+^ signaling plays a crucial role in governing apicomplexan motility; yet, the mechanisms underlying Ca^2+^ mobilization from intracellular stores in these parasites remain unclear. In *Plasmodium*, the necessity of ICM1 for Ca^2+^ mobilization raises the question of whether this mechanism is conserved in other apicomplexans. Investigation into the orthologs of *Plasmodium* ICM1 in *T. gondii* revealed a differing requirement for ICM proteins between the two parasites. This study suggests that *T. gondii* employs ICM-independent mechanisms to regulate Ca^2+^ homeostasis and mobilization. Proteins involved in Ca^2+^ signaling in apicomplexans represent promising targets for therapeutic development.

## INTRODUCTION

Apicomplexan parasites are unicellular eukaryotes that cause a range of life-threatening diseases in humans including malaria, babesiosis, cryptosporidiosis, and toxoplasmosis (1). Malaria is a devastating febrile illness caused by various species of *Plasmodium*, most notably *P. falciparum* and *P. vivax* in humans (2). Malaria is the deadliest apicomplexan disease, resulting in 608,000 deaths from 249 million cases in 2022 (3). Resembling a mild form of malaria, babesiosis is also due to red blood cell infection by an apicomplexan parasite (4). *Babesia microti* (*B. microti*) and *B. divergens* cause human babesiosis, which can be fatal in elderly or splenectomized patients (5, 6). Cryptosporidiosis, caused by *Cryptosporidium* parasites, manifests as a diarrheal disease affecting the small intestine, claiming tens of thousands of children’s lives annually (7). *Toxoplasma gondii* (*T. gondii*) infects and persists within a staggering 25%–30% of humans worldwide, leading to disseminated illnesses collectively known as toxoplasmosis (8). Toxoplasmosis can be fatal for immunosuppressed individuals or developing fetuses and places any patient at risk for retinal vision loss (9). Although apicomplexans exhibit distinct tropisms and pathophysiologies, lytic parasite growth is the primary source of pathogenesis in these organisms (10). Hence, gaining a comprehensive understanding of the mechanisms underlying the progression of these parasites through their lytic cycles will uncover potential molecular targets for the development of next-generation therapies.

The lytic cycle, best dissected in *T. gondii*, unfolds in five sequential steps: attachment to a host cell, host cell invasion, formation of the parasitophorous vacuole (PV), intracellular replication, and finally, host cell egress (11). Attachment is mediated by parasite and host surface proteins/glycoproteins (12). Once attached, parasites sequentially secrete organelles called micronemes and rhoptries (13) to embed a ring-like invasion complex (i.e., moving junction) into the host cell plasma membrane that acts as a portal for parasite entry (14). A surface adhesin-linked actin-myosin motor, termed the glideosome, provides the locomotive force for invasion and other motile processes (11). As the parasite invades, the PV membrane (PVM) is formed from the host plasma membrane stripped of host proteins. The PVM shields the parasite from the hostile cytosolic environment and serves as the gatekeeper for effector export and nutrient acquisition (15–17). A single parasite can generate numerous offspring through asexual replication within the PV (10). At the end of the replication process, parasites secrete specialized microneme proteins (i.e., perforin-like proteins) and enhance their motility to facilitate egress from both the PV and host cell (18). Consequently, the apicomplexan lytic cycle not only amplifies the parasite burden but also induces tissue destruction and inflammation. Emerging evidence indicates that the lytic cycle is not a passive process, but rather, it is orchestrated by environmental cues and signal transduction cascades within the parasites.

Apicomplexans have adapted multiple second messenger signaling pathways to rapidly modulate motility for cell-to-cell transit or static replication (19–23). Purine cyclic nucleotides (cGMP and cAMP) and ionic calcium (Ca^2+^) signaling pathways are central regulators of apicomplexan motility but are utilized in distinct ways. In general, external signals stimulate parasite guanylate cyclase(s) (GCs) to produce cGMP from GTP (24–34). Accumulation of cGMP in the parasite cytosol activates protein kinase G (PKG), the only known cGMP effector in apicomplexans (35). Activated PKG stimulates Ca^2+^ mobilization for microneme secretion and motility (36–42). Cytosolic Ca^2+^ activates Ca^2+^-binding proteins that regulate microneme secretion (e.g., calcium-dependent protein kinases, vesicle fusion machinery) and motility (e.g., calmodulins) (43–45). The role of cAMP signaling has diverged throughout apicomplexan evolution. In *Plasmodium*, cAMP signaling, in conjunction with the protein kinase A catalytic subunit (PKAc), collaborates with cGMP and Ca^2+^ to facilitate invasion (21, 46–50). Conversely, in *T. gondii*, PKAc1 serves to inhibit motility post-invasion by negatively modulating Ca^2+^ (51, 52). However, in both scenarios, it is evident that cyclic nucleotide and Ca^2+^ signaling play essential roles in regulating timely motility within the Apicomplexa phylum.

Recent studies have shed light on the connection between cyclic nucleotide signaling and Ca^2+^ mobilization in apicomplexans. Ca^2+^ is stored within various organelles, including the endoplasmic reticulum, mitochondria, acidocalcisomes, and plant-like vacuolar compartments (19, 53). The canonical eukaryotic Ca^2+^ release channel, known as IP_3_R, acts as a receptor for the second messenger inositol (1, 5, 6)-trisphosphate (IP_3_). While apicomplexans encode the enzymes necessary for IP_3_ synthesis and mobilize Ca^2+^ in response to IP_3_, apicomplexan IP_3_Rs have not yet been identified (54). In *Plasmodium*, it is thought that PKG regulates IP_3_-induced Ca^2+^ by modulating phosphoinositol metabolism (55). Furthermore, a newly identified interactor and potential substrate of *Plasmodium* PKG, known as ICM1, has been found to play a crucial role in Ca^2+^ mobilization in response to cGMP agonists in both *P. berghei* and *P. falciparum* (56). The ICM1 proteins found in *Plasmodium* possess 11 transmembrane (TM) domains and exhibit limited similarity to amino acid transporters, cation transporters, or IP_3_ receptors (56). Further investigations are required to ascertain whether ICM1 proteins function as authentic calcium channels, where they localize, and how they are regulated.

In search of proteins important for Ca^2+^ mobilization in other apicomplexan parasites, we identified and characterized two ICM-like proteins in *T. gondii* (TGGT1_305470; TGGT1_309910). Phylogenetic analysis supported a shared ancestry between apicomplexan ICM-like proteins. Both ICM-like proteins were expressed and detectable in tachyzoites. Unexpectedly, the two ICM-like proteins were amenable to conditional knockdown and/or genetic deletion at no discernible impact on tachyzoite fitness. This result contrasted with a previous genome-wide disruption screen in tachyzoites that indicated both genes to be fitness conferring (57). Furthermore, simultaneous loss of TgICM1-L and TgICM2-L did not compromise parasite viability, motility, or Ca^2+^ mobilization. This study broadens our understanding of the repertoire, localization, and function of ICM-like proteins in apicomplexans and underscores significant evolutionary distinctions in Ca^2+^ mobilization between *T. gondii* and *Plasmodium*.

## RESULTS

### Identification and conservation of putative apicomplexan ICM proteins

**Fig 1:**
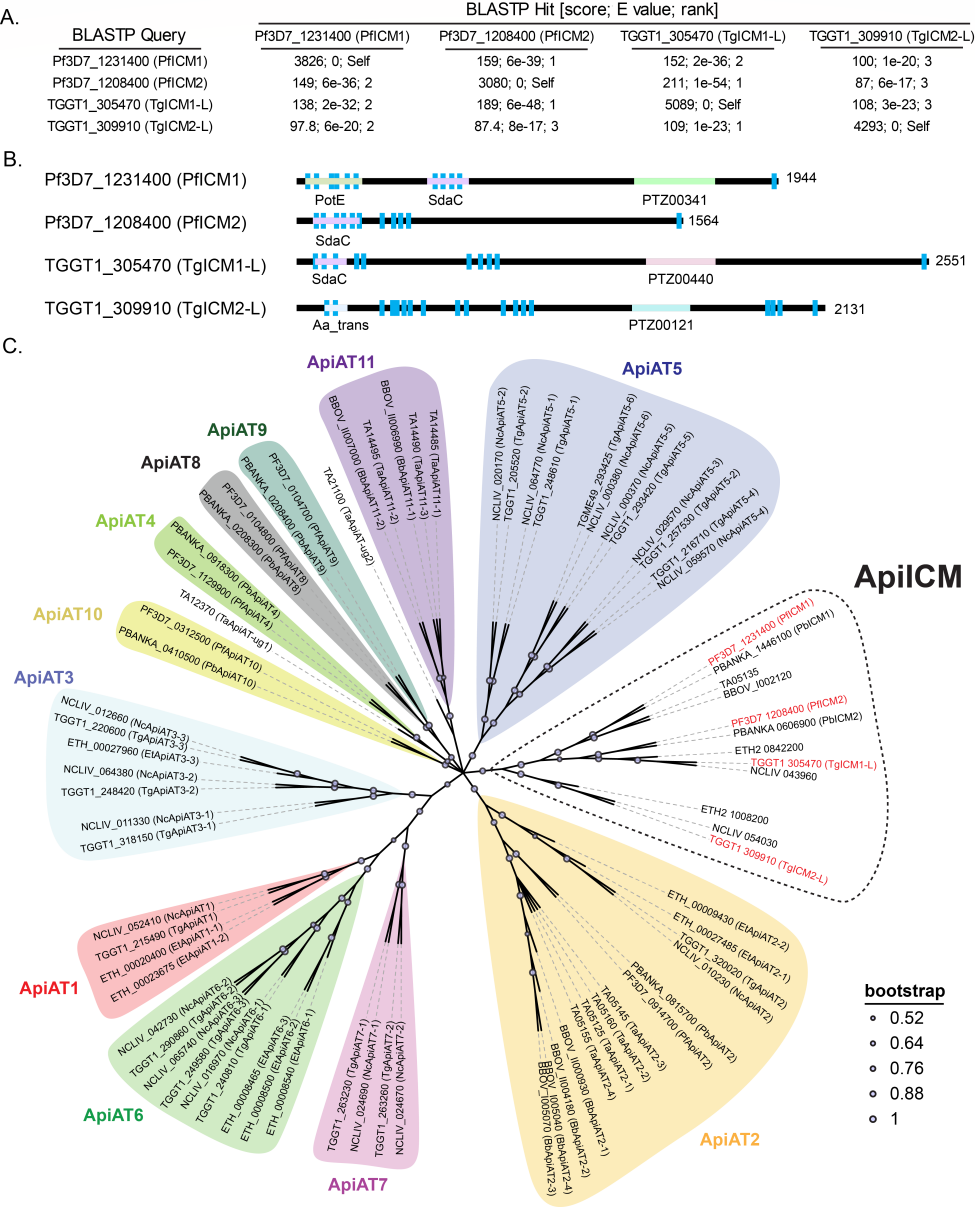
Identification of ICM-Like proteins in *Toxoplasma gondii.* (**A**) Reciprocal BLASTP searches to identify orthologs of *P. falciparum* ICM1 in *T. gondii* (cutoff E < 1e-10). (**B**) NCBI conserved domains and TM domains identified by TOPCONS. SdaC, PotE, Aa_trans are associated with AA transport. (**C**) ApiICM and ApiAT sequences were aligned with MUSCLE and a consensus maximum likelihood tree was constructed with 200 bootstraps. The annotated ApiAT clades matched those published in reference (59).

*Plasmodium* ICM1 is important for Ca^2+^ mobilization in *P. falciparum* and *P. berghei* (56). To search for the existence of an orthologous ICM1 in *T. gondii*, we conducted a BLASTP search using the PfICM1 (Pf3D7_1231400) protein sequence against the *T. gondii* GT1 proteome. Two proteins, TGGT1_305470 (E value: 2e-36) and TGGT1_309910 (E value: 1e-20), were identified as putative ICM-like proteins (Fig. 1A). Reciprocal BLASTP searches of TGGT1_305470 (TgICM1-L) and TGGT1_309910 (TgICM2-L) sequences against the *P. falciparum* and *T. gondii* GT1 proteomes found no other ICM-like proteins, yet revealed a second putative ICM protein in *P. falciparum*: Pf3D7_1208400 (PfICM2) (Fig. 1A). PfICM2 has been demonstrated to be dispensable in *P. falciparum* asexual blood stages based on a genome-wide transposon mutagenesis screen but its role in other life stages or Ca^2+^ transport is not known (58). The BLASTP E value scores were very similar between the four proteins no matter which sequence was queried, suggesting that may share a common ancestor.

To determine whether the four ICM proteins from *P. falciparum* and *T. gondii* share similar domain architectures, we searched for transmembrane domains using TOPCONS (60) and conserved domains using NCBI CD search (61). PfICM1, PfICM2, and TgICM1-L had 10–11 TM domains positioned at relatively similar increments (Fig. 1B). Conversely, 18 TM domains were found in TgICM2-L, suggesting a distinct evolutionary origin, or indicating that it has undergone significant divergence under selective pressure (Fig. 1B). Interestingly, all four proteins had non-specific hits of one or more amino acid transporter/permease domains (i.e., PotE, SdaC, Aa_trans) but not Ca^2+^ transporters (Fig. 1B). Considering the role of ICM1 in *Plasmodium* Ca^2+^ mobilization as documented in previous research (56), it is plausible that these identified proteins may signify a novel class of Ca^2+^ transporters that have evolved from ancient amino acid transporters.

Since the ICM proteins had partial signatures of amino acid transporters, we reconstructed the apicomplexan amino acid transporter (ApiAT) phylogenetic tree (59) with ICM sequences from the same representative apicomplexans (identified by BLASTP). The apicomplexan ICM (ApiICM) proteins formed a distinct outgroup from the 11 ApiAT clades (Fig. 1C), suggesting they may fulfill a related role. However, it should be noted that most of the designated ApiATs have not been investigated for function and may serve other roles as well (59). Within the ApiICM clade, three subclades emerged. The PfICM1 subclade included representatives from *Theileria* (TA05135) and *Babesia* (BBOV_I002120) (Fig. 1C). The PfICM2 subclade included representatives from *T. gondii* (TgICM1-L), *Eimeria* (ETH2_0842200), and *Neospora* (NCLIV_043960) forming subclade with PfICM2 that did not contain representatives from *Theileria* or *Babesia* (Fig. 1C). The third subclade was more divergent, containing representatives from *T. gondii* (TgICM2-L), *Eimeria* (ETH2_1008200), and *Neospora* (NCLIV_054030) (Fig. 1C). These findings indicate that ApiICMs are semi-conserved in Apicomplexa and have diverged from the ApiATs.

### Role of TgICM1-L in tachyzoites

**Fig 2:**
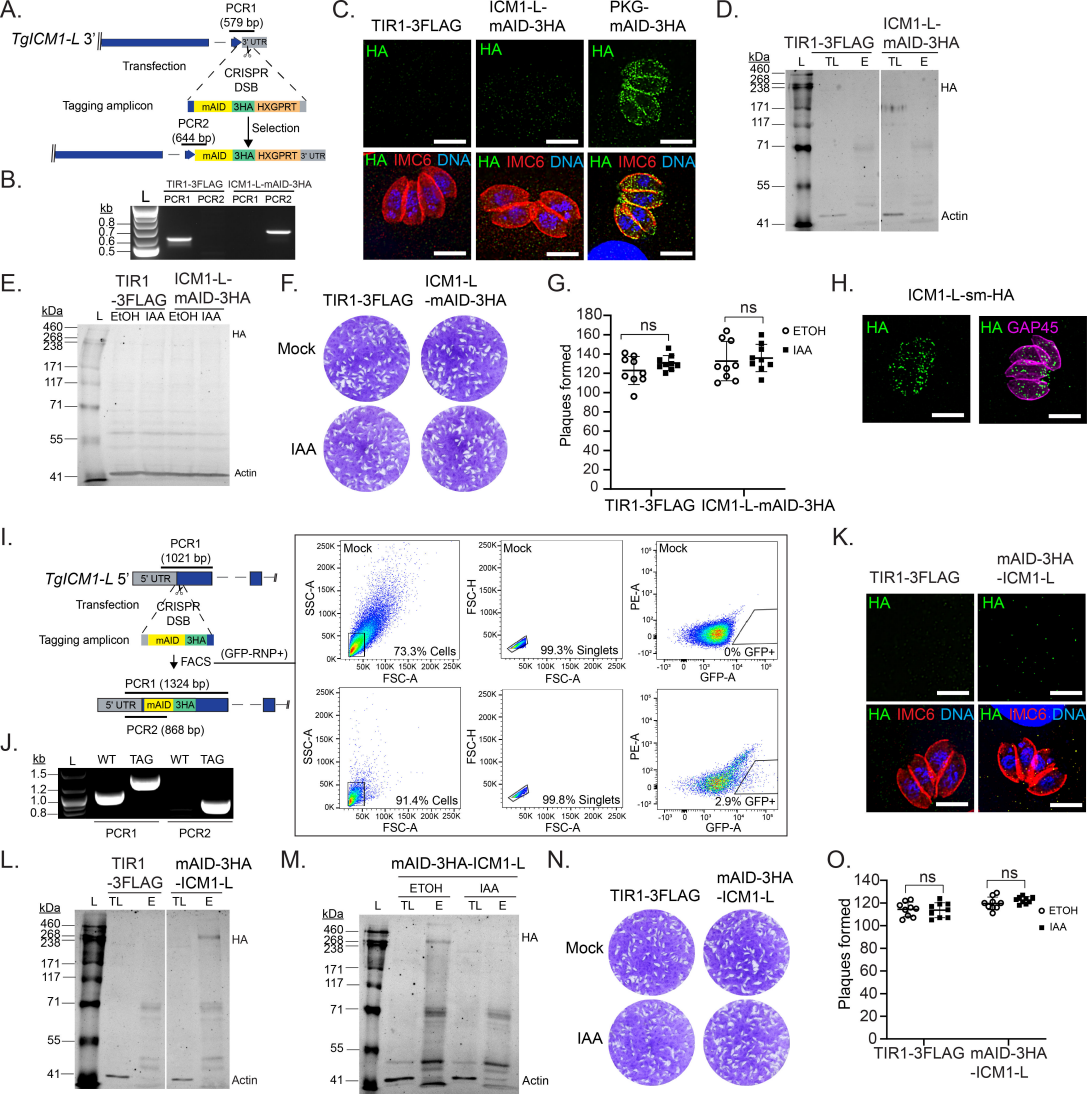
Expression, localization, and conditional knockdown of TgICM1-L in tachyzoites (**A**) Strategy for C-terminal tagging TgICM1-L with mAID-3HA in RH TIR1-3FLAG. (**B**) Diagnostic PCRs from gDNA showing 3′ integration of mAID-3HA into TgICM1-L. (**C**) ICM1-L-mAID-3HA expression in tachyzoites is not detectable by super-resolution microscopy. Scale bar = 5 µm. (**D**) ICM1-L-mAID-3HA enriched by immunoprecipitation (IP) is not detectable by immunoblotting. (**E**) ICM1-L-mAID-3HA expression by immunoblotting following parasite treatment with IAA or the vehicle (ETOH) for 48 h was below the threshold needed to confirm conditional knockdown. (**F and G**) Plaque formation by 200 tachyzoites treated with IAA or the vehicle (ETOH) for 7 days. (**F**) Representative images. (**G**) Mean plaque number (*N* = 3, *n* = 9) +/-SD. n.s, not significant, unpaired two-tailed Student’s *t*-test. (**H**) IF microscopy of ICM1-L-smHA parasites confirms ICM1-L expression in tachyzoites. Scale bar = 5 µm. (**I**) Strategy for N-terminal tagging TgICM1-L with mAID-3HA in RH TIR1-3FLAG using FACS to select for GFP-RNP-transfected parasites. FACS gates defined with mock-transfected parasites. (**J**) Diagnostic PCRs from gDNA showing 5′integration of mAID-3HA into TgICM1-L. (**K**) mAID-3HA-ICM1-L expression in tachyzoites is not detectable by super-resolution microscopy. Scale bar = 5 µm. (**L**) mAID-3HA-ICM1-L enriched by IP is detectable by immunoblotting. (**M**) Confirmation of mAID-3HA-ICM1-L knockdown following treatment with IAA or EtOH for 48 h using IP and immunoblotting. (**N and O**) Plaque formation by 200 tachyzoites treated with IAA or the vehicle (ETOH) for 7 days. (**N**) Representative images. (**O**) Mean plaque number (*N* = 3, *n* = 9) ±SD. n.s, not significant, unpaired two-tailed Student’s *t*-test.

A genome-wide CRISPR disruption screen in *T. gondii* provided evidence supporting a role for TgICM1-L in tachyzoites, indicated by its fitness conferring score of −2.84 (57). For reference, the verified essential kinase, TgPKG (TGGT1_311360), received a fitness score of −2.15 (57). To investigate the function of TgICM1-L in *T. gondii*, we employed a conditional knockdown approach based on our initial hypothesis that TgICM1-L might resist deletion. In the auxin receptor line RH TIR1-3FLAG (41), we tagged TgICM1-L with mAID-3HA at its C-terminus using CRISPR/Cas9 genome editing (Fig. 2A). The resulting TgICM1-L-mAID-3HA line was validated by diagnostic genomic PCR and amplicon sequencing, which confirmed flawless tag integration (Fig. 2B). However, attempts to detect the expression of TgICM1-L-mAID-3HA by immunofluorescence assay (IFA) using super-resolution microscopy (Fig. 2C) or by immunoprecipitation (IP) with immunoblotting (Fig. 2D) proved unsuccessful. Consequently, we were unable to confirm the expression, or verify the knockdown, of the tagged protein in the absence or presence of auxin by immunoblotting (Fig. 2E). We reasoned that TgICM1-L-mAID-3HA may still function at levels below the limit of detection, so we tested whether conditional loss of this protein affects tachyzoite fitness using a plaque assay. However, no significant differences in the apparent size or number of plaques were observed between vehicle- and auxin-treated parasites (Fig. 2F and G). This suggests that TgICM1-L is either dispensable or that its C-terminus is not amenable to auxin-induced degradation (e.g., proteolytically removed). To enhance detection sensitivity, we tagged TgICM1-L with spaghetti monster HA (smHA), a high-affinity tag consisting of 10 copies of the HA epitope (62). Fortunately, we were able to detect TgICM1-L-smHA by IFA (Fig. 2H), indicating that the C-terminal mAID-3HA fusion was likely expressed, but at levels below the limit of detection (Fig. 2C through E). The staining pattern of TgICM1-L-smHA resembled cytosolic puncta (Fig. 2H). Since the C-terminal TgICM1-L knockdown yielded inconclusive results due to detection issues, we repositioned the mAID-3HA to the N-terminus of TgICM1-L using marker-less CRISPR-Cas9 genome editing. Transfected parasites (Cas9-GFP^+^) were selected by fluorescence-assisted cell sorting (FACS) and cloned by limiting dilution (Fig. 2I). After subcloning, flawless tag integration was confirmed by diagnostic PCR and amplicon sequencing (Fig. 2J). Once again, we were unable to detect the mAID-3HA fusion by IFA with super-resolution microscopy (Fig. 2K). Using IP with immunoblotting, we successfully detected mAID-3HA-ICM1-L expression close to the predicted size of 274 kDa (Fig. 2L) and confirmed knockdown within 48 h of IAA treatment (Fig. 2M). Surprisingly, conditional loss of TgICM1-L had no effect on tachyzoite plaque formation (Fig. 2N and O), suggesting that TgICM1-L is either dispensable or refractory to complete knockdown with auxin. Despite predicted essentiality (57), conditional depletion of TgICM1-L in tachyzoites did not produce obvious defects in lytic growth (Fig. 2F, G, N, and O).

**Fig 3:**
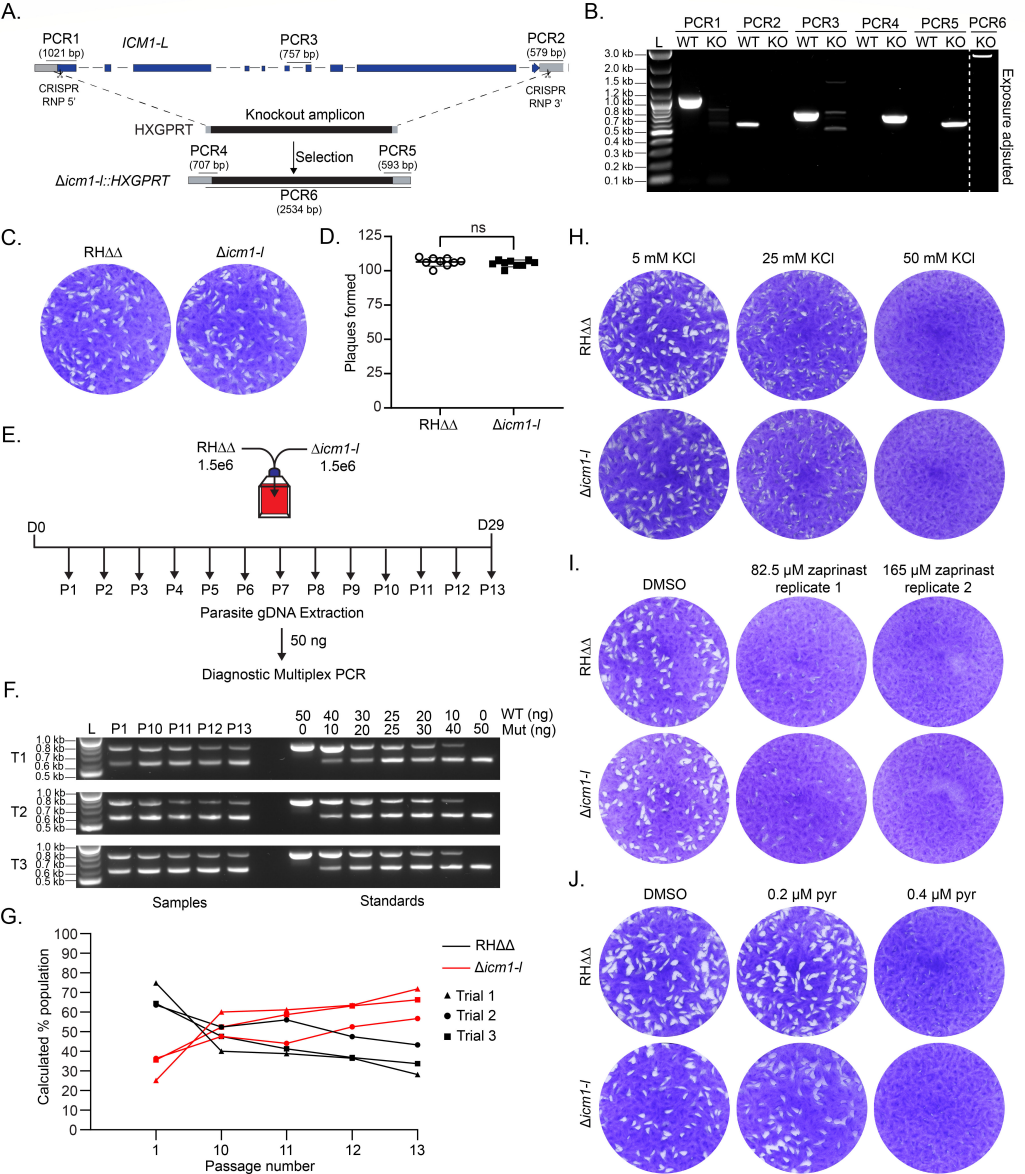
Deletion of TgICM1-L does not negatively impact tachyzoite fitness (**A**) TgICM1-L KO strategy showing diagnostic PCR positions. (**B**) Validation of TgICM1-L deletion by diagnostic PCR. WT = RH*Δku80Δhxgprt*, RH*ΔICM1-L*. (**C and D**) Plaque formation by 200 RH*Δku80Δhxgprt* or RH*ΔICM1-L* parasites at 7 days in HFFs. (**C**) Representative images. **D**) Mean plaque number (*N* = 3) ±SD. (**E–G**) Growth competition assay between RH*Δku80Δhxgprt* and RH*ΔICM1-L* parasites. (**E**) Equal starting numbers of parasites were co-cultured together in HFF T-25s and passaged as needed for 29 days. gDNA samples were collected at each passage for PCR. (**F**) Diagnostic multiplex PCR using 50 ng of gDNA template from co-culture or standard samples. WT = internal ICM1-L 757 bp amplicon. Mut = internal HXGPRT 578 bp amplicon. (**G**) Percentage of RH*Δku80Δhxgprt* and RH*ΔICM1-L* over 29 days based on the ratio of gDNA calculated from the standard curve for each trial. (**H–J**) Plaque formation by 200 RH*Δku80Δhxgprt* and Δ*ICM1-L* parasites at 7 days in HFFs in the presence of increasing KCl (**H**), zaprinast (**I**), or pyrimethamine (**J**).

Using a dual CRISPR/Cas9 ribonucleoprotein (RNP) approach, we replaced *TgICM1-L* with an *HXGPRT* drug marker in RHΔ*hxgprt*Δ*ku80* parasites (Fig. 3A). Multiple diagnostic PCRs (Fig. 3B) and nanopore sequencing (data not shown) confirmed complete loss of *TgICM1-L*. Genetic deletion of *TgICM1-L* did not significantly alter plaque formation (Fig. 3C and D). To determine whether TgICM1-L has a subtle role in tachyzoite fitness, we performed a competitive growth assay between Δ*icm1-l* and its parent RHΔ*hxgprt*Δ*ku80* (Fig. 3E through G). Equal numbers of mutant and parent parasites were co-cultured together in HFFs and passaged/sampled for 29 days as needed (Fig. 3E). Using a diagnostic multiplex PCR strategy, we detected increasing amounts of mutant gDNA and decreasing amounts of parent gDNA over time in three independent trials (Fig. 3F). On average, we calculated that 65% of the co-cultures were mutant after 13 passages (Fig. 3G). These experiments indicate that loss of TgICM1-L does not impair tachyzoite growth under standard growth conditions *in vitro* and may instead impart a slight fitness advantage.

We next examined whether TgICM1-L deletion increases sensitivity to various stress conditions associated with Ca^2+^ mobilization based on the role of ICM1 in *Plasmodium*. First, we assessed parasite sensitivity to extracellular potassium (K^+^). Eukaryotic cells maintain cytosolic K^+^ at relatively high levels compared to their extracellular environment (63). Intracellular parasites can detect reductions in host cell K^+^, such as from rupture of the host plasma membrane, to mobilize Ca^2+^ for egress (64, 65). Consistent with this, increasing the K^+^ concentration in tissue culture media blocks parasite egress (64). We hypothesized that elevating K^+^ concentration in the media would also impair tachyzoite plaque formation by depriving the parasites of a natural signal for Ca^2+^ elevation and egress. We observed equal plaque formation between Δ*icm1-l* and WT tachyzoites in 5 mM K^+^ (normal growth), 25 mM K^+^ (~50% growth), and 50 mM K^+^ (no growth) (Fig. 3H). This result suggests that TgICM-L is individually dispensable for K^+^ sensing for Ca^2+^-dependent egress. Elevated cGMP is also important for Ca^2+^ mobilization and egress, which is strongly induced by the phosphodiesterase inhibitor zaprinast (37, 39, 40). Prolonged exposure to zaprinast disrupts the apicomplexan lytic cycle leading to parasite death (40, 66, 67). WT and Δ*icm1-l* parasites were equally sensitive to sublethal doses of zaprinast, suggesting that TgICM1-L is individually dispensable for basal- and zaprinast-induced cGMP and downstream Ca^2+^ signaling (Fig. 3I).

Conditional and unconditional loss of TgICM1-L did not significantly impact tachyzoite fitness (Fig. 2 and 3), despite having an essential-like fitness score from a genome-wide CRISPR screen (57). In the previous screen, the transfected sgRNA plasmid library was stably selected with pyrimethamine. We hypothesized that the loss of TgICM1-L might have inadvertently sensitized parasites to pyrimethamine, giving the appearance that TgICM1-L is essential. However, our subsequent plaque assay revealed that both WT and Δicm1-l parasites exhibited equal sensitivity to lethal and sublethal doses of pyrimethamine (Fig. 3J). It remains unclear why CRISPR/Cas9-targeted insertions/deletions (indels) in TgICM1-L are detrimental to tachyzoite growth when it can be readily knocked down (Fig. 2) or knocked out (Fig. 3). In either case, it is possible that TgICM2-L may compensate for loss of TgICM1-L in *T. gondii* suggesting potential functional redundancy within the ICM-like protein family.

### Cooperation of TgICM1-L and TgICM2-L in tachyzoites

**Fig 4:**
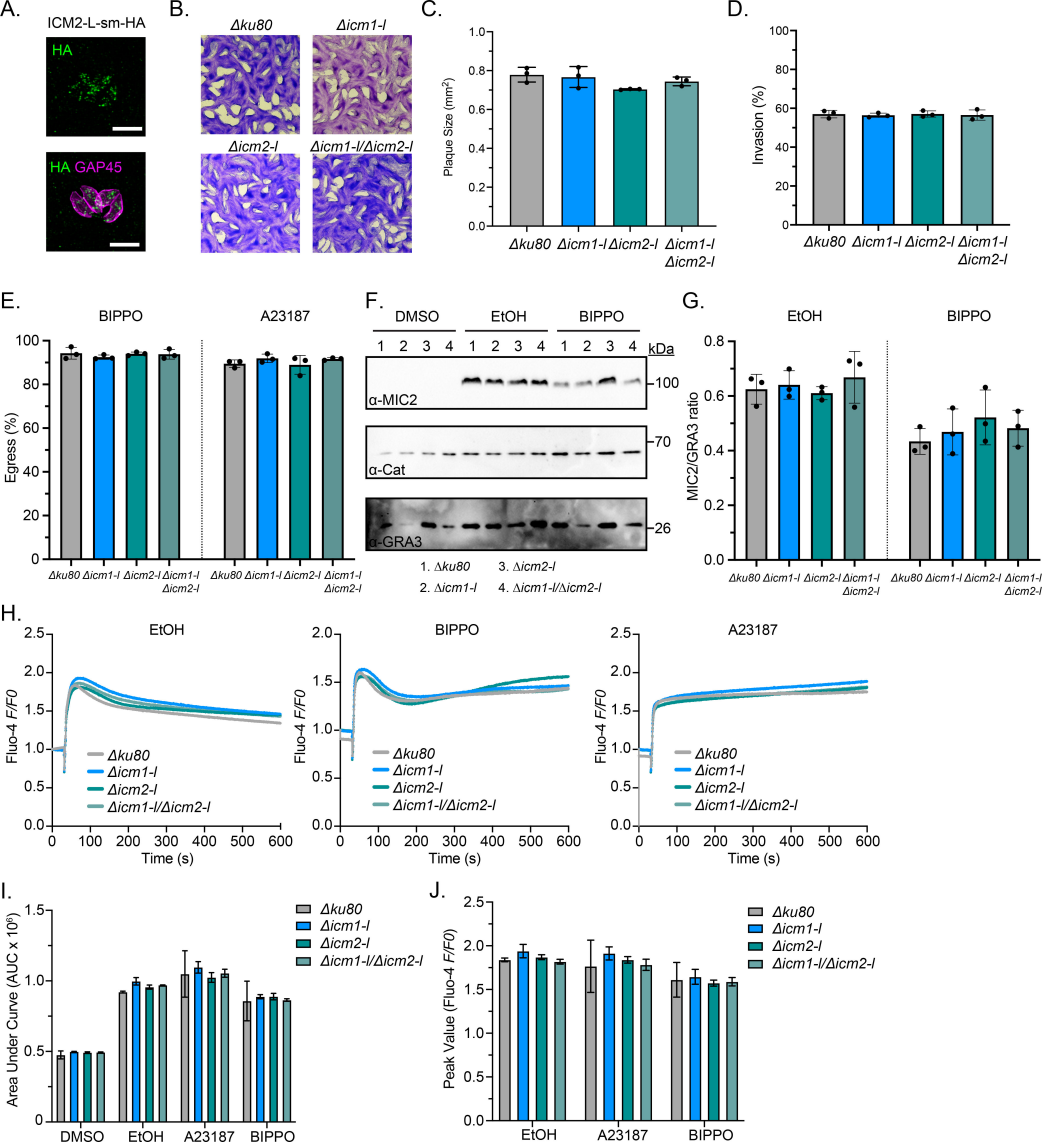
TgICM1-L and TgICM2-L are dispensable for tachyzoite growth, motility, and Ca^2+^ mobilization (**A**) IF microscopy of ICM2-L-smHA parasites confirms TgICM2-L expression in tachyzoites. Scale bar = 5 µm. (**B and C**) Plaque formation by tachyzoites at 7 days in HFFs. (**B**) Representative images. (**C**) Mean area number (*N* = 3) ±SEM. (**D**) Invasion assay showing the percentage of invaded tachyzoites in HFFs. (**E**) Egress assay showing the percentage of egressed parasite vacuoles in HFFs following treatment with BIPPO or A23187. (**F–G**) Microneme secretion assay of tachyzoites stimulated with DMSO, EtOH, or BIPPO. (**F**) Representative immunoblots of excreted secreted antigen fractions. (**G**) The ratio of secreted MIC2 to secreted GRA3 as quantified by immunoblotting (*N* = 3), no significant differences were measured. (**H–J**) Fluo-4 calcium reporter assay showing tachyzoite intracellular calcium changes in response to DMSO, EtOH, BIPPO, and A23187. (**H**) Time-resolved calcium changes in tachyzoites. Traces shown are the mean of 9 replicates per line. (**I**) Area under the curve (AUC) measurements. (**J**) Peak calcium responses. No significant differences were observed.

To confirm TgICM2-L expression in tachyzoites, we appended a C-terminal smHA tag to the *TgICM2-L* gene in RHΔ*hxgprt*Δ*ku80* using CRISPR/Cas9 genome editing. TgICM2-L-smHA appeared as cytosolic puncta (Fig. 4A) as observed for TgICM1-L-smHA (Fig. 2H). We used CRISPR/Cas9 genome editing to delete TgICM2-L individually, and in combination with TgICM1-L, and confirmed the edits by Nanopore long-reads sequencing. Loss of TgICM1, TgICM2, or both did not significantly affect plaque formation (Fig. 4B and C), suggesting that orthologs of *Plasmodium* ICM1 are dispensable for *T. gondii* fitness. To further investigate the role of TgICM1-L and TgICM2-L on infectivity, we compared host cell invasion and egress between parent and mutant parasites. We found parasites lacking TgICM1-L, TgICM2-L, or both were able to invade host cells normally (Fig. 4D). These mutants also egressed from host cells normally in response to cGMP elevation (BIPPO) and Ca^2+^ elevation (A23187) (Fig. 4E). Since invasion and egress both require micronemal proteins, we performed microneme secretion assays. Loss of TgICM1, TgICM2, or both had no significant impact on microneme secretion in response to common secretagogues (Fig. 4F and G). Since *Plasmodium* ICM1 is important for Ca^2+^ mobilization, we tested whether TgIMC1-L and/or TgICM2-L participate in Ca^2+^ mobilization in *T. gondii*. We loaded mutant and parent parasites with Fluo-4 AM, a fluorescent Ca^2+^ indicator, and then stimulated the parasites with known Ca^2+^ agonists including EtOH, BIPPO, and A23187. By tracking changes in cytosolic Ca^2+^ over time, we determined that loss of TgICM1-L, TgICM2-L, or both does not alter the magnitude, kinetics, or resolution of Ca^2+^ mobilization in the parasites (Fig. 4H through J). Taken together, *T. gondii* and *Plasmodium* appear to utilize distinct genes for Ca^2+^ mobilization to control motility.

## DISCUSSION

Apicomplexans mobilize Ca^2+^ from intracellular stores following PKG activation to regulate microneme secretion and motility (37–41). In *Plasmodium*, PKG is believed to trigger Ca^2+^ mobilization by increasing IP_3_ production (55), thereby activating an unidentified IP_3_-sensitive Ca^2+^ channel and through an unknown mechanism involving ICM1 phosphorylation (56). While IP_3_ signaling has been demonstrated in multiple apicomplexans (54, 68–70), it is unclear whether ICM1 is conserved and essential throughout the phylum, and if so, whether it acts as an IP_3_ receptor. ICM1 lacks canonical domains found in calcium channels such as IP_3_R, sarcoendoplasmic reticulum calcium ATPase (SERCA), voltage-dependent Ca^2+^ channels (VDCCs), and transient receptor potential (TRP) channels. Instead, ICM1 and its orthologs have partial fragments of PotE and SdaC domains. In bacteria, PotE is a putrescine transporter which mediates putrescine import by proton symport and putrescine efflux by putrescine:ornithine antiport (71). *P. falciparum* can import putrescine and spermidine, but there are no obvious polyamine-specific transporters encoded in the *P. falciparum* genome (72). Also found in bacteria are SdaC proteins, which are L-serine:proton symporters (73). Apicomplexans exhibit a notable absence of PotE and SdaC orthologs, yet they possess a robust genetic repertoire for amino acid transport, with a total of 11 recognized ApiAT clades (59).

Through comparative genomics and phylogenetic analysis, we identified and classified orthologs of ICM1 in Apicomplexa. Interestingly, apicomplexan ICM proteins exhibit partial signatures of amino acid transporters/permeases. Phylogenetic analysis showed that ApiICMs formed a distinct outgroup from the designated ApiAT clades casting doubt on their ability to transport amino acids or polyamines and suggesting a plausible unique role in calcium transport.

To further elucidate this enigmatic gene family and determine whether apicomplexans utilize similar mechanisms to mobilize Ca^2+^ for motility, we characterized two ICM-like proteins in *T. gondii*. As observed in *Plasmodium* (56), the detection of ICM1-like proteins in *T. gondii* proved challenging, suggesting that they are low-abundance proteins. Our initial characterizations of TgICM1-L and TgICM2-L involved determining their expression and localization in tachyzoites. For TgICM1-L, we were unable to detect N- or C-terminal mAID-3HA tags by immunoblotting or IFA. Prior transcript profiling across two tachyzoite replication cycles showed peak TgICM1-L transcript expression in the G1 phase and ranked in the 56.92%ile of genes expressed in asynchronous culture (74). These data indicate that TgICM1-L is transcribed in tachyzoites but is likely a low-abundance protein. Similarly, PbICM1-3HA was undetectable in *Plasmodium* gametocytes and schizonts by immunoblotting and IFA (56), further supporting a model whereby ICM proteins function at low levels. To maximize our chances of detecting TgICM1-L and TgICM2-L, we utilized the spaghetti monster HA (smHA) epitope tag that was specifically designed to detect low-abundance proteins (62). Using the smHA tag, we were able to visualize TgICM1-L and TgICM2-L by IFA, appearing as cytosolic puncta within tachyzoites. These subcellular distributions partially agree with a HyperLOPIT screen for protein localization in tachyzoites, which placed TgICM1-L in the nucleolus and TgICM2-L in the endoplasmic reticulum (75).

Prior to this study, functional analyses of TgICM1-L and TgICM2-L were limited to a genome-wide CRISPR/Cas9 knockout screen performed in tachyzoites (57). This powerful screen reported fitness scores of −2.84 for TgICM1-L and −0.5 for TgICM2-L. Ranging from −6.89 to 2.96, a gene’s fitness score is highly predictive of dispensability based on benchmarks set by known essential and dispensable genes (57). Genes with fitness scores less than 0 are predicted to contribute to tachyzoite fitness, with scores proportional to gene dispensability. For example, TgPKG, a known essential kinase, has a fitness score of −2.15 while TgUPRT, a known dispensable enzyme, has a fitness score of 0.43 (57). Based on fitness scores, we hypothesized that TgICM1-L is indispensable, and TgICM2-L is dispensable but important in tachyzoites. Surprisingly, loss of TgICM1-L, by conditional knockdown or knockout, did not affect tachyzoite plaque formation, raising a discrepancy between the gene’s fitness score and the observed fitness of the null mutant. Similarly, the loss of TgICM2-L by knockout also did not recapitulate its fitness score based on the null mutant’s observed fitness. Furthermore, pairwise deletion of TgICM1-L and TgICM2-L had no obvious impact on tachyzoite fitness, indicating that their dispensability is not due to functional redundancy with one another. Approach-based limitations could explain inconsistencies between pooled library CRISPR/Cas9 KO screens and single gene mutations. False-positive identification of essential genes from CRISPR/Cas9 KO screens may occur when sgRNAs have off-targets, target repetitive genomic elements, or possibly through gain of function truncations (76–78). While pooled screens take care to limit transfections to ≤1 sgRNA plasmid per cell, >1 sgRNA plasmid may also cause false-positive essential phenotypes. Finally, we considered the possibility that loss of TgICM1-L, in combination with the sgRNA plasmid selection with pyrimethamine in the CRISPR/Cas9 screen (57), could produce a synthetic lethal phenotype if TgICM1-L had a role in folate metabolism. However, we found that mutants lacking TgICM1-L were equally sensitive to pyrimethamine treatment. Therefore, it remains unclear how TgICM1-L and TgICM2-L resembled fitness-conferring genes based on pooled library CRISPR/Cas9 KO screening.

Collectively, TgICM1-L and TgICM2-L were both amenable to deletion individually and in tandem. Mutants lacking TgICM1-L and TgICM2-L displayed normal growth, invasion, egress, and Ca^2+^ mobilization. Contrastingly, in *Plasmodium*, Ca^2+^ mobilization is mediated by ICM1, a protein that is phosphorylated in a PKG-dependent manner and is essential for both asexual blood stages and transmission stages (56). The functional differences of ApiICMs between species may be partially explained by their phylogeny. Our phylogenetic analysis grouped TgICM1-L in the same subclade as PfICM2, a paralog of PfICM1 previously shown to be dispensable in *P. falciparum* asexual blood stages (58). We inferred that the closest ICM1 orthologs are restricted to hematazoan blood parasites including *Plasmodium*, *Babesia*, and *Theileria*. Similarly, true ICM2-L orthologs appear to be restricted to coccidian parasites. Therefore, we conclude that the ApiICM family has undergone expansion, contraction, and specialization throughout apicomplexan evolution.

In conclusion, our results point to a model in which apicomplexans have evolved specialized genes to mobilize Ca^2+^ for motility. Unlike *Plasmodium*, the ApiICM gene family is dispensable in *T. gondii* tachyzoites, indicating that other protein(s) are responsible for Ca^2+^ mobilization. Since cGMP signaling is upstream of Ca^2+^ signaling, identification of PKG substrates may reveal novel proteins responsible for regulating Ca^2+^ homeostasis and mobilization in *T. gondii*. Our results also raise important technical considerations when investigating low-abundance proteins and proteins anticipated to be essential based on high-throughput reverse genetic screens. We recommend that gene essentiality should be validated by single-gene genetic approaches, with careful consideration for the growth conditions in which essentiality is assessed.

## MATERIALS AND METHODS

### Sequence analysis

Annotated genomic, transcript, and protein sequences were downloaded from https://veupathdb.org/veupathdb/app. Transmembrane domains within ICM protein sequences were predicted using TOPCONS, a consensus of OCTOPUS, PHILIUS, PolyPhobius, SCAMPI, and SPOCTOPUS algorithms (60). Protein domains and features were predicted using the NCBI conserved domain search against the CDD v3.20-59693 PSSMs database with an Expect Value threshold of 0.01 (79). The predicted transmembrane domains and features were drawn to scale (primary aa length) using Adobe Illustrator (Adobe, Inc.).

### Phylogenetic analysis

The 66 ApiAT representatives (59) were aligned with 12 ApiICM representatives by MUSCLE in MEGA11. All positions with less than 80% site coverage were eliminated, that is, fewer than 20% alignment gaps, missing data, and ambiguous bases were allowed at any position (partial deletion option). There were 474 aligned positions in the final data set. The evolutionary history was inferred using the Maximum Likelihood method and JTT matrix-based model. The percentage of trees in which the associated taxa clustered together is shown next to the branches based on 200 bootstrap replicates.

### Parasite and host cell culture

*T. gondii* tachyzoites were maintained in human foreskin fibroblast (HFF) monolayers at 37°C, 5% CO_2_ in D3 or D10 medium: Dulbecco’s Modified Eagle’s Medium (Gibco) supplemented with 3% or 10% fetal bovine serum (Gibco), 10 mM glutamine (Gibco), and 10 µg/mL gentamicin (Gibco). Cell lines were routinely assessed for *Mycoplasma* contamination by PCR using a Myco-Sniff Mycoplasma PCR Detection Kit (MP Biomedicals). All parasite lines used or generated in this study are listed in Table S1.

### Antibodies

Mouse anti-HA.11 (Clone 16B12) was purchased from BioLegend. Rat anti-HA (Clone 3F10) was purchased from Roche. Mouse anti-IMC6 was provided by Peter Bradley, Ph.D. (University of California Los Angeles). Rabbit anti-GAP45 and rabbit anti-TgCatalase were produced in the lab of Dominique Soldati-Favre, Ph.D. (University of Geneva). Mouse anti-MIC2 and mouse anti-TgGRA3 were provided by J.F. Dubremetz, Ph.D. (Université de Montpellier). Rabbit anti-TgActin was provided by David Sibley, Ph.D. (Washington University in St. Louis). Goat secondary antibodies conjugated to infrared (IR) dyes and Alexa Fluor dyes were purchased from LI-COR and Invitrogen, respectively.

### Plasmids

All plasmids generated in this study were created by Q5 Site-Directed Mutagenesis (New England Biolabs) of existing plasmids or HiFi Gibson Assembly (New England Biolabs) of linear dsDNA fragments. Plasmid sequences were confirmed by Sanger sequencing (Genewiz) and mapped using SnapGene v5.2.4 (GSL Biotech). All plasmids used in this study are listed in Table S2.

### Oligonucleotides and PCR

All synthetic ssDNA and ssRNA oligonucleotides were synthesized by Integrated DNA Technologies and are listed in Table S3. Genomic DNA was extracted for PCR using a Monarch Genomic DNA Purification Kit (New England Biolabs). Q5 Polymerase (New England Biolabs) was used for cloning and tagging amplicon PCRs. Taq polymerase (New England Biolabs) was used for diagnostic PCRs.

### Parasite transfections

One or two million tachyzoites were transfected by electroporation in a P3 buffer using a 4D-nucleofector (Lonza) using FI-158 pulse code. Parasites were allowed to recover in HFFs for 24 hours prior to selection.

### Generation of TgICM1-L conditional knockdown mutants

RH TIR1-3FLAG tachyzoites were used for tagging TgICM1-L with mAID-3HA as described (80). For c-terminal tagging, 2 × 10^6^ freshly harvested TIR1-3FLAG tachyzoites from the RH strain were transfected with a pSAG1:Cas9-GFP, U6:sgTgICM1-L 3′ UTR plasmid (9 µg) to create a double-strand break in the 3′ UTR of TgICM1-L near the translation stop codon. This plasmid was co-transfected with corresponding 40 bp homology arm-flanked mAID-3HA, HXGPRT amplicon (1 µg). For N-terminal tagging, 2 × 10^6^ freshly harvested RH TIR1-3FLAG tachyzoites were co-transfected with a CRISPR/Cas9 RNP (0.125 µg/µL) containing the 5′ gRNA sequence to the first exon of TgICM1-L and a corresponding 40 bp homology arm-flanked mAID-3HA amplicon (1 µg). mAID-3HA-ICM1-L-transfected parasites were selected by FACS using a FACSAria Fusion Flow Cytometer (BD Biosciences) for Cas9-GFP + expression. Following selection, parasites were subcloned by limiting dilution in 96-well plates, and clones identified by visual confirmation of single plaques were screened by diagnostic PCR on ChemiDoc MP imaging system (Bio-Rad), analyzed and adjusted for brightness and contrast using Image Lab software (Bio-Rad). Correct integration of the mAID-3HA tag in the C-terminal or N-terminal of TgICM1-L was confirmed by Sanger sequencing.

### Generation of TgICM1-L-smHA and TgICM2-L-smHA lines

RHΔ*hxgprt*Δ*ku80* was co-transfected with Cas9 expressing plasmid containing a gRNA targeting at the 3′UTR region of TgICM1-L-smHA and TgICM2-L-smHA and a 30 bp homology arm containing smHA and chloramphenicol acetyltransferase (CAT) drug selection cassette. Following selection, parasites were subcloned by limiting dilution.

### Generation of TgICM1-L and TgICM2-L knockouts

Δ*icm1-l* parasites were generated by transfecting RHΔ*hxgprt*Δ*ku80* parasites with CRISPR/Cas9 RNPs (IDT Cas9-GFP loaded with 3′ and 5′ *ICM1-L* targeting gRNAs) and an *HXGPRT* cassette (40 bp homology arms) to replace *ICM1-L*. Following selection, parasites were subcloned by limiting dilution, and confirmed by diagnostic PCR and amplicon sequencing. These parasites were used for experiments related to Fig. 2 and 3. A separate Δ*icm1-l* line was also created by replacing *ICM1-L* in RHΔ*hxgprt*Δ*ku80* with chloramphenicol acetyltransferase (CAT) (30 bp homology arms) using a CRISPR/Cas9 plasmid strategy. Following selection, parasites were subcloned by limiting dilution. These parasites were used for experiments related to Fig. 4. To generate Δ*icm2-l,* and Δ*icm1-l/* Δ*icm2-l* mutants, CRISPR/CaS expressing plasmid carrying guide-RNA targeting at the 3′UTR of TgICM2-L was co-transfected with 30 bp homology arm with DHFR-TS drug marker cassette in RHΔ*hxgprt*Δ*ku80* and RHΔ*hxgprt*Δ*ku80*; Δ*icm1-l*::CAT strain, respectively. Correct integration of the two drug markers to generate the aforementioned strains was confirmed by Nanopore Long-Reads Sequencing (Oxford Nanopore Technologies plc).

### Knockdown of mAID-3HA protein fusions

Knockdowns were performed as described (80). RH TIR1-3FLAG or mAID-3HA-tagged tachyzoites cultivated in HFFs in D10 medium were treated with 0.5 mM 3-indoleacetic acid (auxin; IAA) (Sigma Aldrich) prepared in 100% ethanol (Pharmco) or vehicle alone (0.0789% wt/vol ethanol final concentration) and incubated at 37°C, 5% CO_2_ prior to protein detection and/or phenotypic analysis.

### Detection of epitope-tagged proteins by indirect immunofluorescence microscopy

HFF monolayers grown on 12-mm glass coverslips (Electron Microscopy Sciences) were infected with tachyzoites and fixed with 4% formaldehyde (Polysciences) in phosphate-buffered saline (PBS), permeabilized with 0.1% Triton X-100 (MP Biomedicals), blocked with 10% normal goat serum (Gibco) in PBS, labeled with mouse anti-HA primary antibody (1:1,000), and rabbit anti-IMC6 (1:2,000) or anti-GAP45 (1:10,000) primary antibody for 1 h and then washed three times with PBS. Subsequently, HA-tagged proteins were labeled with anti-mouse IgG monoclonal secondary antibody conjugated to Alexa Fluor 488 (Thermo Fisher Scientific) diluted 1:2,000 for 1 h, and parasites labeled with anti-rabbit IgG monoclonal secondary antibody conjugated to Alexa Fluor 594 (Thermo Fisher Scientific) diluted 1:2,000 for 1 h. Parasite nucleus was stained using Hoechst 33258 diluted 1:2,000. Coverslips were washed five times with PBS, rinsed with water, and mounted on glass slides with Prolong Gold antifade reagent (Invitrogen). Images were acquired with a Nikon CSU-W1 SoRa dual spinning disk confocal microscope at 100× oil objective running SoRa mode for a total 280× magnification, analyzed with a NIS-Element software (Ninon, Inc.), and processed with Adobe Photoshop 2023 V24.7 (Adobe Systems Inc., United States). Alternatively, images representing the localization of smHA-tagged ICM1-L and ICM2-L proteins were obtained with Leica Stellaris 5 confocal microscope using 63× oil objective followed by processing by software ImageJ (version 2.1.0).

### Immunoprecipitation

HFFs cultivated in T-150 flasks were infected with 4 × 10^7^ TgICM1-L-mAID-3HA or mAID-3HA-TgICM1-L tachyzoites for 48 h. Following, cultures were scraped, syringe-lysed three times with a 25-gauge needle to release remaining intracellular parasites, and centrifuged at 800 × *g*, 18°C, 10 min. Approximately 5 × 10^8^ parasites were lysed in 6 mL lysing buffer (10 mM K_2_HPO_4_, 150 mM NaCl, 5 mM EDTA, 5 mM EGTA, 0.2% sodium deoxycholate, 1% Triton X-100, pH 7.4) supplemented with Pierce Protease Inhibitor Cocktail for 1 h on ice. Lysates were syringe lysed three times with a 25-gauge needle, the total lysate sample was collected, the remaining lysate was centrifuged at 3,200 × *g*, 4°C, 50 min and the supernatant incubated with 0.25 mg of anti-HA magnetic beads overnight in a shaker at 4°C. Magnetic beads were then collected by centrifugation at 500 × *g*, 4°C, 10 min, and the elution fraction was resuspended in 100 µL cold PBS for immunoblotting as described below.

### SDS-PAGE and immunoblotting

For routine detection of proteins, tachyzoite pellets were lysed in an equal volume of 2× Laemmli buffer (81) containing 20 mM dithiothreitol (DTT) (Thermo Fisher Scientific). All other protein samples were mixed 4:1 with 5× Laemmli buffer containing 50 mM DTT. Proteins were separated on 8% polyacrylamide gels (Bio-Rad) by SDS-PAGE and wet-blotted onto nitrocellulose membranes. The membranes were rinsed with PBS containing 0.1% Tween-20 (PBS-T), and then blocked with PBS-T containing 5% (wt/vol) fat-free powdered milk (blocking buffer). Membranes were probed with rat anti-HA primary antibody (1:500), and rabbit anti-Tgactin (1:7,000) diluted in blocking buffer, then washed three times with PBS-T. The membranes were then incubated with goat anti-rat 800 CW (1:5,000) and goat anti-rabbit 680 RD (1:5,000) secondary antibodies (LI-COR) diluted in blocking buffer, then washed five times with PBS-T. Membranes were imaged on a ChemiDoc MP Imaging System. Gels and membrane blots were analyzed using Image Lab software (Bio-Rad).

### Plaque assays

Freshly egressed tachyzoites were harvested, counted on a hemocytometer, and inoculated onto confluent HFF monolayers growing in six-well or twelve-well plates containing D3 medium (100-200 parasites/well). To determine the effect of conditional knockdown of mAID-3HA-tagged proteins on *T. gondii* fitness, wells were treated with 0.5 mM IAA or 0.0789% wt/vol ethanol (vehicle). Plates were left undisturbed for 7 days in a 37°C, 5% CO_2_ incubator. Plaque formation was assessed by counting zones of clearance on formaldehyde-fixed, crystal violet-stained HFF monolayers. Each stained plate was scanned with a high-definition digital scanner (Epson) to obtain representative images and for plaque area analysis.

### Competition assays

RHΔ*hxgprt*Δ*ku80* and Δ*icm1-l* tachyzoites were harvested, scraped, syringe lysed three times with a 25-gauge needle, centrifuged at 500 × *g*, 18°C, 10 min, resuspended in D3 and counted. 1.5 × 10^6^ parasites from each line were inoculated and co-cultured into HFF flasks and passed every 2 or 3 days for 29 days. Parasite gDNA was isolated in each passage and 50 ng of DNA was used as a template for diagnostic multiplex PCR. Amplicons were separated by gel electrophoresis, imaged with a ChemiDoc MP (BIO-RAD), and densitometry was performed using Image Lab (BIO-RAD).

### Invasion assays

Freshly harvested tachyzoites were used for infection of HFF monolayers seeded on coverslips and centrifuged at 1,000 *g* for 1 min. Infected HFFs were incubated at 37°C for 30 min, followed by 10 min fixation with paraformaldehyde-glutaraldehyde (PFA/Glu) and 5 min neutralization with PBS/0.1M glycine at room temperature. The fixed samples were blocked using 5% PBS-BSA for 20 min at room temperature and stained by anti-SAG1 antibody for 1 h. After staining, slides were fixed with 1% formaldehyde for 7 min and permeabilized with PBS/0.2% Triton-X100 for 20 min. Permeabilized samples were stained with anti-GAP45 antibody and visualized by corresponding secondary antibodies. At least 100 parasites per condition were counted to determine the invasion ratio. Data presented in this study were from three biological replicates.

### Egress assays

HFF monolayers infected by tachyzoites were incubated for 30 h at 37°C and incubated with serum-free DMEM buffer containing DMSO (0.2%), BIPPO (10 µM), or A23187 (5 µM). After induction, the cultures were fixed by PFA/Glu for 10 min and neutralized by PBS/0.1M glycine for 5 min. To assess the egress event, the parasites and PVs were visualized by antibodies against TgGAP45 and TgGRA3, respectively. Egress ratio were determined by raptured PV/total PV (>200 were counted in each replicate). The data presented in the study were obtained from three individual replicates.

### Microneme secretion assays

Freshly egressed parasites were washed twice in warm DMEM media pelleted and resuspended in serum-free warm DMEM containing 10 µM BIPPO or 5 µM Ca^2+^ ionophore A23187. Resuspended parasites were incubated at 37°C for 15 min to allow microneme secretion and pelleted at 2,000 *g* for 10 min to separate pellet/supernatant (ESA) fraction. An additional centrifugation at 2,000 g for 5 min was conducted to remove the remaining cell debris from ESA. All samples were subjected to immunoblot analysis using anti-TgMIC2, anti-Tgcatalase, and anti-TgGRA1 antibodies.

### Calcium measurements

Parasites were harvested after freshly released and washed three times using intracellular (IC) buffer composed of 5 mM NaCl, 142 mM KCl, 2 mM EGTA, 1 mM MgCl_2_, 5.6 mM glucose, and 25 mM HEPES-KOH at pH 7.2. Following washing, 2 × 10^7^ parasites were resuspended in 100 µL IC buffer supplemented with 5 µM Fluo-4 AM and allowed to pre-load the dye for 1 h at room temperature in the dark. After preloading, parasites were washed once with IC buffer to remove excess dye and then resuspended for further analysis. Subsequently, 20 µL (4 × 10^6^) of the resuspended parasites was injected into each well of a 384-well plate. Upon starting the baseline read, 40 µL of a solution containing corresponding compounds (final concentration: DMSO 2%, EtOH 2%, BIPPO 10 µM, or A23187 5 µM) was added. The signal was detected using a 488 nm Blue Laser with a 530/30 filter, with baseline recordings taken for 60 seconds followed by readings for 10 min to generate the curve.

### Statistical analysis

Data were graphed and analyzed for statistical significance using GraphPad Prism v 9 (GraphPad Software) using a two-tailed Student’s *t*-test for pairwise comparisons of normally distributed data. Error bars represent the standard deviation or standard error of the mean as indicated in the figure legends. Differences between means were considered statistically significant when *P* was <0.05 and designated with one or more asterisks above the graphed data. Non-significant differences between means (*P* > 0.05) were labeled with “ns” in the figures.

## SUPPLEMENTAL MATERIAL

Table S1 spectrum.01229-24-s0001.docx Strains used.

Table S2 spectrum.01229-24-s0002.docx Plasmids used.

Table S3 spectrum.01229-24-s0003.docx Oligonucleotides used.

ASM does not own the copyrights to Supplemental Material that may be linked to, or accessed through, an article. The authors have granted ASM a non-exclusive, world-wide license to publish the Supplemental Material files. Please contact the corresponding author directly for reuse.
