# Supplementary material for: Orthologs of Plasmodium ICM1 are dispensable for Ca2+ mobilization in Toxoplasma gondii
Source: Microbiol Spectr. 2024 Aug 20;12(10):e01229-24. doi: 10.1128/spectrum.01229-24 (PMC11448412; doi:10.1128/spectrum.01229-24)
Supplement: Table S1 — Strains used. [file spectrum.01229-24-s0001.docx]

**Table S1**. *T. gondii* strains used in this study.

| **Shorthand Strain Name** | **Genotype** |
| --- | --- |
| RHΔ*hxgprt*Δ*ku80* | RHΔ*hxgprt*Δ*ku80* |
| RH TIR1-3FLAG | RHΔ*hxgprt*Δ*ku80*; *TUB1*:*TIR1-3FLAG, SAG1*:*CAT* |
| RH ICM1-L-mAID-3HA | RHΔ*hxgprt*Δ*ku80*; *TUB1*:*TIR1-3FLAG, SAG1*:*CAT*; *ICM1-L-mAID-3HA*, *DHFR-TS*:*HXGPRT* |
| RH mAID-3HA-ICM1-L | RHΔ*hxgprt*Δ*ku80*; *TUB1*:*TIR1-3FLAG, SAG1*:*CAT*; *mAID-3HA*-*ICM1-L* |
| RHΔ*icm1-l* | RHΔ*hxgprt*Δ*ku80*; Δ*icm1*-*l*::*DHFR-TS*:*HXGPRT* |
| RHΔ*icm1-l* | RHΔ*hxgprt*Δ*ku80*; Δ*icm1*-*l*::*TUB1*:*CAT* |
| RHΔ*icm2-l* | RHΔ*hxgprt*Δ*ku80*; Δ*icm2-l*::*DHFR-TS*:*DHFR-TS** |
| RHΔ*icm1-l/*Δ*icm2-l* | RHΔ*hxgprt*Δ*ku80*; Δ*icm1*-*l*::*TUB1:CAT;* Δ*icm2-l*::*DHFR-TS*:*DHFR-TS** |
| RH ICM1-L-smHA | RH*ΔhxgprtΔku80*; *ICM1-L-sm-HA, TUB1*:*CAT* |
| RH ICM2-L-smHA | RHΔ*hxgprt*Δ*ku80*; *ICM2-L-sm-HA*, *TUB1*:*CAT* |
