# Supplementary material for: Orthologs of Plasmodium ICM1 are dispensable for Ca2+ mobilization in Toxoplasma gondii
Source: Microbiol Spectr. 2024 Aug 20;12(10):e01229-24. doi: 10.1128/spectrum.01229-24 (PMC11448412; doi:10.1128/spectrum.01229-24)
Supplement: Table S2 — Plasmids used. [file spectrum.01229-24-s0002.docx]

**Table S2.** Plasmids used in this study.

| **p#** | **Plasmid Name** | **Description** | **Usage** | **Source** |
| --- | --- | --- | --- | --- |
| p1 | p*TUB1:YFP-mAID-3HA, DHFR-TS:HXGPRT* | *YFP-mAID-3HA* fusion driven by a minimal *TgTUB1* promoter with an *TgHXGPRT* drug selectable marker. | PCR template for generating TgICM1-L*-mAID-3HA*, mAID-3HA-ICM1-L tagging amplicons and Δ*icm1*-l knockout amplicon | Brown et al. 2017 |
| p2 | p*SAG1:Cas9-GFP, U6:_gRNA* | Linear *SpCas9-GFP*, *gRNA* (no protospacer) destination vector | Linear *Cas9-GFP, gRNA* (no protospacer) destination vector for inserting TgICM1-L protospacer by ssDNA (60mer) HiFi assembly. CRISPR plasmid targeting *Tg*ICM1-L 3’ UTR for C-terminal tagging | Shen et al. 2014 |
| P3 | pLIC-smHA-CAT | Spaghetti monster-HA (10x HA) fusion encoding a Chloramphenicol acetyltransferase (CAT) drag selectable marker | PCR template for generating TgICM1-L-sm-HA and TgICM2-L-sm-HA | Triana et.al  2018 |
| p3 | pSAG1:Cas9-GFP, U6:sgTgICM1-L 3’ UTR | *Streptococcus pyogenes* *Cas9* fused to *GFP* driven by a *TgSAG1* promoter and *CRISPR sgRNA targeting TgICM1-L 3’ UTR* driven from a *Pol III TgU6* promoter | Co-transfection with *TgICM1-L-mAID-3HA, DHFR:HXGPRT* amplicon for CRISPR/Cas9 tagging C-terminus of *TgICM1-L*. | This work |
| p3 | pSAG1:Cas9-GFP, U6:sgTgICM1-L 5’ | *Streptococcus pyogenes* *Cas9* fused to *GFP* driven by a *TgSAG1* promoter and *CRISPR sgRNA targeting TgICM1-L 5’ UTR* driven from a *Pol III TgU6* promoter | Co-transfection with amplicon containing chloramphenicol (CAT) selection marker to generate ICM1-L knock-out line. | This work |
| p3 | pSAG1:Cas9-GFP, U6:sgTgICM1-L 3’ | *Streptococcus pyogenes* *Cas9* fused to *GFP* driven by a *TgSAG1* promoter and *CRISPR sgRNA targeting TgICM1-L 3’ UTR* driven from a *Pol III TgU6* promoter | Co-transfection with amplicon containing CAT selection marker and smHA epitope tag to generate TgICM1-L-sm-HA | This work |
| p3 | pSAG1:Cas9-GFP, U6:sgTgICM2-L 5’ | *Streptococcus pyogenes* *Cas9* fused to *GFP* driven by a *TgSAG1* promoter and *CRISPR sgRNA targeting TgICM2-L 5’ UTR* driven from a *Pol III TgU6* promoter | Co-transfection with amplicon containing DHFR-TS selection marker to generate ICM2-L knock-out and ICM1-L/ICM2-L double knock-out lines. | This work |
| p3 | pSAG1:Cas9-GFP, U6:sgTgICM2-L 3’ | *Streptococcus pyogenes* *Cas9* fused to *GFP* driven by a *TgSAG1* promoter and *CRISPR sgRNA targeting TgICM1-L 3’ UTR* driven from a *Pol III TgU6* promoter | Co-transfection with amplicon containing CAT selection marker and smHA epitope tag to generate TgICM2-L-sm-HA | This work |
