# Supplementary material for: Orthologs of Plasmodium ICM1 are dispensable for Ca2+ mobilization in Toxoplasma gondii
Source: Microbiol Spectr. 2024 Aug 20;12(10):e01229-24. doi: 10.1128/spectrum.01229-24 (PMC11448412; doi:10.1128/spectrum.01229-24)
Supplement: Table S3 — Oligonucleotides used. [file spectrum.01229-24-s0003.docx]

**Table S3.** Oligonucleotides used in this study.

| **O#** | **OligoNT Name** | **Sequence (5’ – 3’)** | **Usage** | **Source** |
| --- | --- | --- | --- | --- |
| O1 | TgICM1-mAID_F | CGCCAGTCTTTTGTACCAAGTGGTTTGGGGCGAGGGCAATGCTAGCAAGGGCTCGGGCTC | Amplify TgICM1-L-mAID-3HA, DHFR-TS:HXGPRT tagging amplicon from pTUB1:YFP-mAID-3HA, DHFR-TS:HXGPRT | This work; IDT |
| O2 | TgICM1-mAID_R | CACATGTAATGCGCGACGCCTGTTTCGAGAGAGTGCAGAGATAGGGCGAATTGGAGCTCC |  | This work; IDT |
| O3 | N-mAID-3HA-ICM1 F | CTTTTTACGAAAACGAGACACCGCAACCATGCGGGACGGAGAGAAGAGCGCGTGTCCTAA | Amplify mAID-3HA-TgICM1-L tagging amplicon from pTUB1:YFP-mAID-3HA, DHFR-TS:HXGPRT | This work; IDT |
| O4 | N-mAID-3HA-ICM1 R | CGGGGCTGGCGAGCGGCGGCAGCAGCCGCTCCCCCAGCGGGGCATAATCTGGAACATCGT |  | This work; IDT |
| O5 | TgICM1-HXGPRT_F | ACTCCTCCTCCACTTTCTTTTTACGAAAACGAGACACCGCCGAGGTCGACGGTATCG | Amplify Δ*icm1-l*, DHFR-TS:HXGPRT knockout amplicon from pTUB1:YFP-mAID-3HA, DHFR-TS:HXGPRT | This work; IDT |
| O6 | TgICM1-HXGPRT_R | TGCAGTGAAGAAATAACAACGTGGTTTCCACGCGAGCAAAGTAAAACGACGGCCAGT |  | This work; IDT |
| O7 | sgTgICM1 5' KO | AACCAUGCGGGACGGACCGCGUUUUAGAGCUAUGCU | *Tg*ICM1-L protospacers used for Alt-R® CRISPR-Cas9 crRNA design for N-terminal tagging and Knock out TgICM1-L | This work; IDT |
| O8 | sgTgICM1 3' KO | GUACCAAGUGGUUUGGGGCGGUUUUAGAGCUAUGCU |  | This work; IDT |
| O9 | Alt-R® CRISPR-Cas9 tracrRNA | Proprietary | Universal 67mer tracrRNA that hybridizes to crRNA to activate the Cas9 enzyme | This work; IDT |
| O10 | sgTgICM1 -3' protospacer | GTAAATGGGGATGTCAAGTTAGACTGCCCCTGCATGTGGGGTTTTAGAGCTAGAAATAGC | *Tg*ICM1-L ssDNA protospacer for HiFi assembly | This work; IDT |
| O11 | TgICM1 WT_F | ATTTAGCTGAATGTCGAAGT | Forward primer for TgICM1-L-mAID-3HA diagnostic PCR 1, PCR 2. Forward primer for Δ*icm1-l* diagnostic PCR 2. Sanger sequencing primer for confirmation of flawless tag integration | This work; IDT |
| O12 | TgICM1 WT_R | CTGACGTTCGCCTTTAACCT | Reverse primer for TgICM1-L-mAID-3HA diagnostic PCR 1. Reverse primer for Δ*icm1-l* diagnostic PCR 2 and PCR 5 | This work; IDT |
| O13 | PCR2 TgICM1_R | CACATCATAGGGATAGCCAGC | Reverse primer for TgICM1-L-mAID-3HA and mAID-3HA-TgICM1-L diagnostic PCR 2. Sanger sequencing primer for confirmation of flawless tag integration | This work; IDT |
| O14 | TgICM1 5' WT_F | AAGGAGTTCGCAGAGGCCTG | Forward primer for mAID-3HA-TgICM1-L diagnostic PCR 1, PCR 2. Forward primer for Δ*icm1-l* diagnostic PCR 1 and PCR 4. Sanger sequencing primer for confirmation of flawless tag integration | This work; IDT |
| O15 | TgICM1 5' WT_R | TGCAGCGTATTCGACTCTCT | Reverse primer for mAID-3HA-TgICM1-L diagnostic PCR 1. Reverse primer for Δ*icm1-l* diagnostic PCR 1. Sanger sequencing primer for confirmation of flawless tag integration | This work; IDT |
| O16 | TgICM1 Exon 6_F | GTACGAAACATGACGCCTTC | Forward primer for Δ*icm1-l* diagnostic PCR 3 | This work; IDT |
| O17 | TgICM1 Exon 7_R | CTCTCTTCCCCTGCAGGTTG | Reverse primer for Δ*icm1-l* diagnostic PCR 3 | This work; IDT |
| O18 | DHFR 5' UTR R | TGCGAACAGCAGCAAGATCGGATC | Reverse primer for Δ*icm1-l* diagnostic PCR 4 | This work; IDT |
| O19 | DHFR 3' UTR F | CGCTGAATCCGTCCAGATCAG | Forward primer for Δ*icm1-l* diagnostic PCR 5 | This work; IDT |
| O20 | PCR 6 v3 F | AAGATGGCTTTCGCTACGCT | Forward primer for Δ*icm1-l* diagnostic PCR 6 | This work; IDT |
| O21 | PCR 6 v3 R | GACGTTCGCCTTTAACCTGC | Reverse primer for Δ*icm1-l* diagnostic PCR 6 | This work; IDT |
| O22 | Guide ICM1-KO | GTCCTGGTTCTCCTCGCTCTGTTTTAGAGCTAGAAATAGC | Forward primer to create the vector pSAG1-Cas9-U6-ICM1-5’ | This work; IDT |
| O23 | ICM1-KO-CAT-F | CCTCCGAGGCTAGCCATCGACGATGCCGATGCGGCCGCTCTAGAACTAG | Forward primer to amplify the doner DNA co-transfected with pSAG1-Cas9-U6-ICM1-5’ to construct ICM1L-KO-CAT | This work; IDT |
| O24 | ICM1-KO-CAT-R | CCGAACCTCTGCAGTCATTTCGTTCGCCCAGGTACCGGGCCCCCCCTC | Reverse primer to amplify the doner DNA co-transfected with pSAG1-Cas9-U6-ICM1-5’ to construct ICM1L-KO-CAT | This work; IDT |
| O25 | Guide ICM2-KO | GCTTCTGAGGAACCTCTACGAGTTTTAGAGCTAGAAATAGC | Forward primer to create the vector pSAG1-Cas9-U6-ICM2-5’ | This work; IDT |
| O26 | ICM2-KO-DHFRTS-F | CCCCATTCCCCCTCGTAGGTGGTGTGCAGCGCGGCCGCTCTAGAACTAG | Forward primer to amplify the doner DNA co-transfected with pSAG1-Cas9-U6-ICM2-5’ to construct ICM2L-KO-DHFRTS and ICM1L-KO-CAT/ ICM2L-KO-DHFRTS | This work; IDT |
| O27 | ICM2-KO-DHFRTS-R | GGAGAACAACTTCCTCGTCGTCTCTGTGCCTCGCGGAAGATCCGATCTTGC | Reverse primer to amplify the doner DNA co-transfected with pSAG1-Cas9-U6-ICM2-5’ to construct ICM2L-KO-DHFRTS and ICM1L-KO-CAT/ ICM2L-KO-DHFRTS | This work; IDT |
| O28 | Guide ICM1-sm-HA | GACACAGGTCCGCCCACATGCGTTTTAGAGCTAGAAATAGC | Forward primer to create the vector pSAG1-Cas9-U6-ICM1-3’ | This work; IDT |
| O29 | ICM1-sm-HA-F | TTGTACCAAGTGGTTTGGGGCGAGGGCAATATGTACCCTTATGATGTGCC | Forward primer to amplify the doner DNA co-transfected with pSAG1-Cas9-U6-ICM1-3’ to construct ICM1-sm-HA | This work; IDT |
| O30 | ICM1-sm-HA-R | TTTCGAGAGAGTGCAGAGACGCCAGACTGCCCGCTGCAAGGCGATTAAGTTG | Reverse primer to amplify the doner DNA co-transfected with pSAG1-Cas9-U6-ICM1-3’ to construct ICM1-sm-HA | This work; IDT |
| O31 | Guide ICM2-sm-HA | GTTGGTGCCTTGAAAGTGGAAGTTTTAGAGCTAGAAATAGC | Forward primer to create the vector pSAG1-Cas9-U6-ICM2-3’ | This work; IDT |
| O32 | ICM2-sm-HA-F | CCTCCCTTTCTTCGATGTCCTGACTCCGACACGATGTACCCTTATGATGTGCC | Forward primer to amplify the doner DNA co-transfected with pSAG1-Cas9-U6-ICM2-3’ to construct ICM2-sm-HA | This work; IDT |
| O33 | ICM2-sm-HA-R | GGAGAACAACTTCCTCGTCGTCTCTGTGCCTCGCTGCAAGGCGATTAAGTTG | Reverse primer to amplify the doner DNA co-transfected with pSAG1-Cas9-U6-ICM2-3’ to construct ICM2-sm-HA | This work; IDT |
